# Phone-Based Parental Support Program for Caries Prevention in Children: A Randomized Controlled Trial

**DOI:** 10.1177/23800844241296054

**Published:** 2024-12-04

**Authors:** I. Brännemo, T. Hasselblad, A. Levinsson, G. Dahllöf, G. Tsilingaridis

**Affiliations:** 1 Division of Pediatric Dentistry, Department of Dental Medicine, Karolinska Institutet, Stockholm, Sweden; 2 Center for Pediatric Oral Health, Stockholm, Sweden; 3 Department of Epidemiology, Biostatistics, and Occupational Health, McGill University, Montreal, QC, Canada; 4 Department of Social Medicine and Public Health, Sahlgrenska Academy, Gothenburg University, Gothenburg, Sweden; 5 Center for Oral Health Services and Research Mid-Norway, TkMidt, Trondheim, Norway

**Keywords:** general anesthesia, dental caries, health inequities, motivational interviewing, pediatric dentistry, preventive dentistry

## Abstract

**Introduction:**

Children referred for comprehensive dental care under general anesthesia, due to severe early childhood caries, have a high risk of continued caries progression in posttreatment years.

**Objectives:**

To assess the effect of a phone-delivered, motivational interviewing–based parental support program on caries recurrence and oral health habits in preschool children treated under general anesthesia for severe early childhood caries.

**Methods:**

The prospective design of this 2-arm randomized clinical trial (allocation ratio 1:1; blinded outcome assessment) comprised 151 patients from pediatric dental departments in the Stockholm region of Sweden. Inclusion criteria were healthy children aged <6 y with early childhood caries who were scheduled for treatment under general anesthesia. Control group parents received standard advice on toothbrushing and sugar reduction. Intervention group parents received planned phone counseling with an oral health coach every other week for 1 y, based on motivational interviewing principles, offered in Arabic, English, Polish, Turkish, and Swedish. The primary outcome was caries progression 1 and 2 y postsurgery, assessed using the International Caries Detection and Assessment System. Secondary outcomes were parent-reported daily toothbrushing and dietary habits.

**Results:**

Sixty-five percent of the control group and 77% of the intervention group experienced caries relapse on at least 1 new surface after 1 y (nonsignificant). At the 2-y follow-up, relapse rates were 53% (control group) and 71% (intervention group; *P* < 0.05) compared with baseline. The intervention group was significantly less likely to engage in adverse oral health behaviors such as snacking on sweets (intervention group, 10%; control group 33%) and sweet drinks (intervention group 9%; control group, 29%) after 1 y. No group differences in daily fluoride toothpaste brushing occurred.

**Conclusion:**

The motivational interviewing–based parental support program improved dietary habits but showed no effect on caries recurrence in children treated under general anesthesia for early childhood caries.

ClinicalTrials.gov NCT02487043

**Knowledge Transfer Statement:**

The findings of this study can assist clinicians, public health leaders, and researchers in tailoring preventative behavior-focused programs for early childhood caries. These results may improve the understanding of how behavioral interventions that involve parents of young children affect caries prevention, highlighting approaches that are less likely to be effective and guiding future efforts toward more promising strategies for high-risk populations.

## Introduction

Dental caries is a common chronic disease in children (Pitts et al. 2017). Its prevalence is skewed, disproportionally affecting socially disadvantaged families and minority populations (Schwendicke et al. 2015). Consequences of early childhood caries (ECC), such as pain and infection, affect the well-being of children and undermine their ability to thrive (Sheiham 2006). Further, ECC sets the child on a disease trajectory with an increasing caries burden later in life (Broadbent et al. 2008).

Young children with severe ECC often require full-mouth rehabilitation under general anesthesia (GA) to accept comprehensive dental care. Despite an often-radical approach, caries recurrence is common in the years following surgery (Almeida et al. 2000; Amin et al. 2010). Treatment involving solely surgical repair and application of pharmacologic agents is seldom sufficient to prevent future decay when negative dental behaviors, such as high exposure to sugar and irregular toothbrushing with fluoride toothpaste, persist (Twetman and Dhar 2015). Parental guidance and upstream action are being increasingly recognized as mitigating caries risk (Tinanoff et al. 2019).

Counseling based on motivational interviewing (MI) has proven useful for other pediatric conditions (Borrelli et al. 2015). For caries prevention, the results are so far mixed but suggest that patient-family–centered interventions (American Academy of Pediatric Dentistry [AAPD] 2013), built on the principals of MI, could be an effective way of achieving changes in parental behavior (Albino and Tiwari 2016).

The present randomized, controlled trial assessed the effect of a phone-delivered, MI-based parental support program on caries recurrence and oral health habits in children treated under GA for ECC.

## Patients and Methods

### Trial Design

The Regional Ethics Review Board in Stockholm approved this prospective, assessor-blinded, 2-arm randomized, controlled trial (daybook No. 2014/1863-31; registration: https://clinicaltrials.gov/, identifier: NCT02487043).

### Participants

#### Recruitment

**Figure 1.**
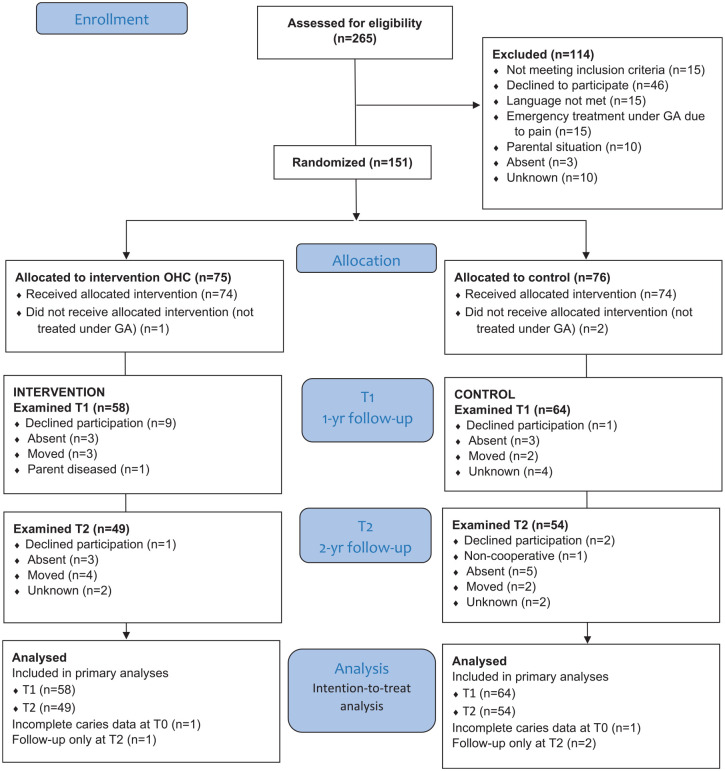
Flow diagram of patients.

Patients were recruited from the pediatric dental departments at Karolinska Institutet and the Public Dental Service in the Stockholm region, Sweden. Inclusion criteria were patients aged <6 y who were scheduled for treatment of ECC under GA. Exclusion criteria included treatment under GA for reasons other than dental caries, parents who could not communicate in any of the offered languages, and children with medical conditions or learning disabilities, as such conditions may present additional health-related and behavioral challenges in maintaining oral health. Figure 1 presents participant flow. All parents signed informed, written consent forms.

#### Randomization

Randomization to 1 of the 2 groups occurred after the principal investigators determined the patient to be eligible and written parental consent was received. Participants were assigned to treatment groups through independent allocation managed by an external party not otherwise involved in the study. A pregenerated list from www.random.org, using block randomization with a 1:1 allocation ratio, was used. Researchers enrolling participants had no access to the allocation sequence, ensuring the integrity of the randomization.

### Intervention

#### Control

Control group participants and their parents met in person with an oral health coach (OHC) prior to randomization and treatment to receive information about and give their written consent to participate in the study. The current oral health situation of the child was discussed, along with standard advice on toothbrushing (twice daily with fluoride toothpaste) and sugar reduction. When needed, an interpreter was made available at the first visit to ensure that full, informed consent was established. After surgery, the control group received standard treatment, including a recall visit to a dental clinic 3 to 6 mo after the procedure. The child’s primary dental team scheduled further recall visits based on need. A 1-y follow-up visit with the OHC was scheduled.

#### Intervention

The intervention group received the same as the control group, plus planned biweekly (i.e., twice monthly) phone counseling with an OHC for 1 y, starting within 2 wk after GA. Counseling consisted of parental support in maintaining positive oral health behaviors at home over time. The intervention was based on principles of family-patient–centered care emphasizing the family as collaborators (AAPD 2013) and was informed by previous studies on MI and telephone counseling in caries prevention (Weinstein et al. 2006; Pukallus et al. 2013). Counseling in Arabic, English, Polish, Turkish, and Swedish was available, striving to be culturally and linguistically sensitive (AAPD 2013).

#### OHC training

The OHCs were 4 dental nurses with experience in pediatric dentistry, recruited from the Public Dental Service, each with differing language skills. They attended an initial 3-d training on ECC prevention, the study protocol, behavior change, and self-management strategies led by a pediatric dentist (I.B.) and a clinical psychologist (T.H.) with previous experience working with MI. The training itself followed MI principles such as building rapport, asking open-ended questions, pros and cons, readiness to change, and strategies for overcoming barriers (Rollnick and Miller 1995). The aim was to explore the individual circumstances and barriers of the family and tailor support according to those needs rather than to give unsolicited advice. The OHCs were trained in change talk through video demonstrations, written information, role-playing, and clinical home assignments. With parental consent, the psychologist gave the OHCs feedback based on the audio recordings of intervention phone calls. The MI treatment integrity (MITI; version 4.1) coding system for MI adherence was used in training (Moyers et al. 2016). Throughout the project, 2 psychologists were available for guidance and support. Group training sessions were held twice a year for group cohesion.

#### Intervention delivery

During training, the OHCs were given a written counseling guide for use in their intervention conversations. The counseling guide included standard information on the structure of MI-based talk, examples of pros and cons, and open-ended questions. During each phone call, the OHC would use a study form to note the time and duration of the call, the main topics discussed, and agreed-upon goals as a support for future contacts. Quarterly, OHCs used importance and confidence rulers to evaluate participants’ perceptions of the significance of changing habits and their confidence in their ability to do so. Participants rated the importance of engaging in their child’s oral health habits (0 = *not important*, 10 = *extremely important*) and their confidence in altering such behaviors (0 = *not confident*, 10 = *extremely confident*). These assessments facilitated the exploration of barriers and the encouragement of change talk with parents (Rollnick and Miller 1995). The strategies were tailored over time for each family.

### Study Measures

The primary outcome measure was the child’s caries progression 1 and 2 y postsurgery, assessed by clinical examinations based on the International Caries Detection and Assessment System (ICDAS; Pitts and Ekstrand 2013). Caries progression was defined as a previously noncavitated tooth or tooth surface developing cavitated caries. The Total Observed Caries Experience (TOCE) index was used to avoid bias due to decayed primary teeth being exfoliated. TOCE is calculated by the total number of teeth with dental caries, treated or untreated, ever observed in a child over time (Ruff 2018).

Secondary outcome measures were oral health–related behaviors such as daily tooth brushing with fluoride toothpaste and dietary habits.

#### Clinical examination

A visual caries assessment was done at baseline (T0, time of GA) and after 1 (T1) and 2 y (T2). The teeth were dried using air or cotton rolls and examined with a mouth mirror under good lighting in a clinical dental setting. When necessary, a toothbrush was used to remove debris. Each surface was scored using the ICDAS 6-grade diagnostic system (Pitts and Ekstrand 2013); noncavitated lesions were graded as 1 at the first visual change in enamel and 2 at a distinct visual change in enamel when wet. Cavitated lesions were graded as 3 for visible localized enamel breakdown, without signs of dentin involvement, and 4 when an underlying dark shadow in the dentin was visible. A distinct cavity with visible dentin involvement was graded as 5 and an extensive distinct cavity with visible dentin involvement as grade 6. The examiners also registered clinical signs of plaque or gingivitis (yes/no).

Seven calibrated pediatric dentists conducted the clinical examinations over a 6-y period. Calibration sessions occurred continuously as new examiners joined or left the study clinics. All examiners attended a 90-min e-learning session on ICDAS (Topping et al. 2008) and a half-day course with lectures and calibration exercises led by a senior examiner (first author I.B., previously described; Brännemo et al. 2021). Reliability was compared with a previously set standard (Anderson et al. 2016). The weighted Cohen’s kappa for interexaminer reliability reached an average of 0.93 (range 0.81–0.98) and for intraexaminer reliability 0.87 (range 0.79–0.95). A written description of the ICDAS criteria with example photographs was appended to all examinations.

#### Questionnaires

**Figure 2.**
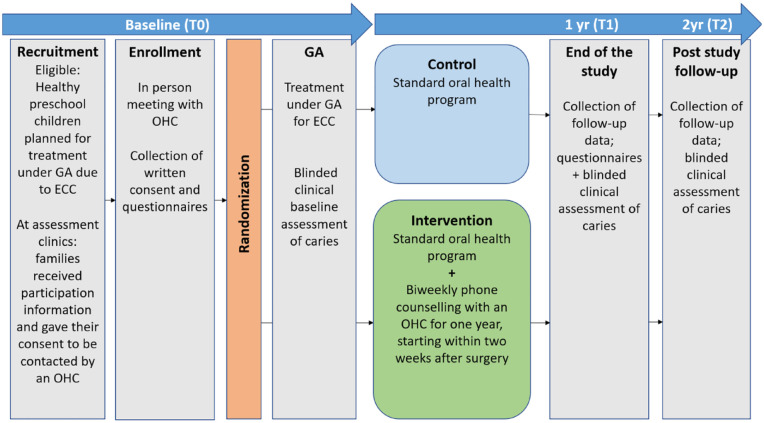
Flowchart of the study. Overview of activities according to the timeline: baseline (T0), 1-y follow-up (T1), and 2-y follow-up (T2) after general anesthesia. Baseline, T1, and T2 activities that are shaded gray include both the intervention and control groups. GA, general anesthesia; OHC, oral health coach; ECC, early childhood caries.

Caregiver-reported measures were collected through questionnaires on sociodemography and oral health habits (reported in this study) as well as parental stress, dental fears, attitudes, and the oral health–related quality of life of the child (to be reported elsewhere). Parents completed the questionnaires at their first meeting with the OHC and again 1 y postintervention. Figure 2 presents an overview of the study activities and timeline.

Families were categorized using the residential area–based caries risk measure in the region of Stockholm (health need area [HNA]). HNA 1 to 2 indicates low caries risk, and HNA 3 to 4 indicates high risk (SLL 2018).

### Blinding

Due to the nature of the intervention, neither participants nor OHCs could be blinded during intervention delivery or questionnaire data collection. Dentists assessing clinical status were blinded to the assigned treatment group. Participants were instructed by the OHC not to mention group assignment to the dentist at clinical examinations. If blinding was broken, the dentists were asked to report this on the clinical examination form.

### Sample Size

Power analyses estimated that a minimum sample size of 130 parent-child dyads would be needed to reach significance, based on the assumption that 10% of the children in the intervention group and 30% of the children in the control group would develop caries after 1 y (Amin et al. 2010). With a significance level of 5% and a power of 80%, 65 patients each would be required in the intervention and control groups. To accommodate for dropout, our goal was to invite 200 families to the study.

### Statistical Analyses

Intention-to-treat analyses were done, excluding participants only when outcome data were unavailable. Patient characteristics at each time point were presented as mean and standard deviations (continuous variables) or count and percentages (categorical variables) for the intervention and control groups. Cross-sectional differences between the intervention and control groups were tested using an independent samples *t* test or a chi-square test. The longitudinal outcome for caries progression (TOCE) was modeled using generalized estimating equations (GEE) with a negative binomial distribution; the longitudinal dichotomous outcome, toothbrushing habits, used logistic GEE. The dichotomous outcome of new caries on at least 1 surface compared with baseline was analyzed using univariable logistic regressions. The significance level was set to α = 0.05. Statistical analyses were done using SPSS (version 27; IBM Corp).

## Results

### Final Population and Background Characteristics

The inclusion rate was lower than expected, and enrollment was closed after 4 y (October 2015 to October 2019); 151 child-parent dyads consented to participate after being found eligible. Three patients were excluded after randomization due to not receiving the planned treatment under GA or providing any follow-up data. Group size was thus 74 patients in each group (Fig. 1). No significant differences in baseline characteristics between groups were observed (Table 1). All children had high levels of caries experience, with an average of 11.7 cavitated teeth (range 4–16) in the control group and 12.2 (range 3–20) in the intervention group. In both groups, more than half of the parents reported that their child frequently consumed sugary drinks and sweet snacks, and about one-third brushed their teeth twice daily with fluoride toothpaste (Table 2). Eighty-two percent of the patients completed the 1-y assessment and 70% the 2-y assessment (Fig. 1). Comparisons of the baseline characteristics of participants with and without data at the follow-ups revealed no significant differences.

**Table 1.** Baseline Characteristics of Patients and Accompanying Adults.

	Controls (*n* = 74)	Intervention (Health Coach) (*n* = 74)	*P* Value (Group Difference)
Age of child (mo)			0.865
Mean (SD)	54.4 (11.89)	54.1 (12.26)	
Sex (child)			0.317
Boy	40 (54.1%)	46 (62.2%)	
Girl	34 (45.9%)	28 (37.8%)	
Age of respondent (y)			0.643
Mean (SD)	34.9 (6.85)	35.5 (7.19)	
Missing data	5 (6.8%)	3 (4.1%)	
Sex (respondent)			0.211
Male	26 (35.1%)	19 (25.7%)	
Female	48 (64.9%)	55 (74.3%)	
Parental background^a^			0.454
Sweden	15 (20.3%)	10 (13.5%)	
Europe (outside Sweden)	14 (18.9%)	19 (25.7%)	
Outside of Europe	45 (60.8%)	45 (60.8%)	
HNA			0.066
1–2	49 (66.2%)	38 (51.3%)	
3–4	25 (33.8%)	36 (48.7%)	

HNA, health need area.

a Based on reported native language. Residential area mapped as high-caries-risk areas (HNA 3–4) or low-caries-risk areas (HNA 1—2).

**Table 2.** Oral Health and Behavioral Variables over Time.

Variables	Baseline		1-y Follow-up		2-y Follow-up	
	Controls (*n* = 74)^a^	Intervention (*n* = 74)^a^	Controls (*n* = 74)^a^	Intervention (*n* = 74)^a^	Controls (*n* = 74)^a^	Intervention (*n* = 74)^a^
TOCE						
Mean (SD)	11.7 (3.62)	12.2 (3.55)	12.8 (3.58)	14.1 (3.39)^b^	13.4 (3.33)	15.0 (3.52)^b^
Missing	1 (1.4%)	1 (1.4%)	10 (13.5%)	16 (21.6%)	20 (27.0%)	25 (33.8%)
Plaque						
No	27 (37.0%)	19 (26.0%)	38 (59.4%)	35 (60.3%)	30 (55.6%)	29 (59.2%)
Yes	46 (63.0%)	54 (74.0%)	26 (40.6%)	23 (39.7%)	24 (44.4%)	20 (40.8%)
Missing	1	1	10	16	20	25
Gingivitis						
No	43 (58.9%)	36 (49.3%)	59 (92.2%)	58 (100%) ^b^	51 (94.4%)	46 (93.9%)
Yes	30 (41.1%)	37 (50.7%)	5 (7.8%)	0 (0%)	3 (5.6%)	3 (6.1%)
Missing	1	1	10	16	20	25
New caries, surface level^c^						
No	—	—	22 (34.9%)	13 (22.8%)	25 (47.2%)	14 (29.2%)^b^
Yes	—	—	41 (65.1%)	44 (77.2%)	28 (52.8%)	34 (70.8%)
Missing			11	17	21	26
Increased TOCE^c^						
No	—	—	26 (41.3%)	15 (26.3%)	18 (34.0%)	6 (12.5%)^b^
Yes	—	—	37 (58.7%)	42 (73.7%)	35 (66.0%)	42 (87.5%)
Missing			11	17	21	26
Tooth brushing 1× daily						
No	32 (43.2%)	31 (41.9%)	14 (24.1%)	14 (24.1%)	—	—
Yes	42 (56.8%)	43 (58.1%)	48 (78.7%)	44 (75.9%)	—	—
Missing	0	0	12	16		
Tooth brushing 2× daily						
No	49 (66.2%)	46 (62.2%)	32 (51.6%)	30 (51.7%)	—	—
Yes	25 (33.8%)	28 (37.8%)	30 (48.4%)	28 (48.3%)	—	—
Missing	0	0	12	16		
Meals/snacks >5						
No	23 (31.1%)	16 (21.6%)	36 (57.1%)	42 (71.2%)	—	—
Yes	51 (68.9%)	58 (78.4%)	27 (42.9%)	17 (28.8%)	—	—
Missing	0	0	11	15	—	—
Sweets for snack						
No	31 (41.9%)	31 (41.9%)	42 (66.7%)	53 (89.8%)^b^	—	—
Yes	43 (58.1%)	43 (58.1%)	21 (33.3%)	6 (10.2%)	—	—
Missing	0	0	11	15	—	—
Sweet drinks						
No	26 (35.1%)	37 (50.0%)	44 (71.0%)	54 (91.5%)^b^	—	—
Yes	48 (64.9%)	37 (50.0%)	18 (29.0%)	5 (8.5%)	—	—
Missing	0	0	12	15	—	—

Daily toothbrushing: child brushes teeth with fluoride toothpaste at least once per day; meals/snacks >5: child has more than 5 meals and snacks per day; sweet drinks: child consumes sweet drinks between meals; sweets for snack: child consumes candy, chocolate, pastries, or chips at least 3 d per week; SD, standard deviation; TOCE, Total Observed Caries Experience.

a Total number of children included in the group, missing reported in table.

b *P* value for difference between groups <0.05.

c Compared to baseline.

### Intervention Delivery

In the intervention group, 62% were able to participate using their reported native language. The remaining 38% (28 parents) participated using Swedish, despite not being native speakers.

Two-thirds (68%) of the intervention group took part in 11 or more phone sessions. Six families participated in at least 20 contacts. The average duration of a phone call was 10 min, with a mean interval of 28.6 d between sessions. Reasons for not implementing biweekly contacts were parent unavailability to speak, parent wanting less contact toward the end of the intervention, changes in family situation, or the OHC on leave.

### Outcomes

#### Caries progression

The intervention group had a significantly higher caries burden (TOCE) at both T1 and T2 as well as a higher caries progression as measured by both an increase in TOCE and new caries on the surface level at T2 compared with T0. More than half of the control group and more than 70% of the intervention group had caries on at least 1 new surface after 2 y (Table 2).

#### Caregiver self-reported dietary and oral health habits

At the 1-y follow-up, intervention group participants were significantly less likely to engage in adverse oral health behaviors such as snacking on sweets and consuming sweet drinks. No group differences in toothbrushing habits were observed, but the clinical examination found significantly less gingivitis in the intervention group compared with the control group (Table 2).

The effect of the intervention after 1 y showed increased mean TOCE score over time but improved toothbrushing habits both once and twice daily in both groups. No significant differences between groups were found (Appendix Table 1).

#### Factors predicting caries development

Univariable logistic regressions were used to explore factors predicting caries progression at T1 and T2. No significant differences between groups were observed for toothbrushing habits or parental background (Appendix Table 2). Sensitivity analyses (not shown) also found no significant between-group differences when group differences in caries burden over time were estimated using models controlling for HNA.

Within the intervention group, none of the studied factors (toothbrushing frequency, parental background, intervention language, phone call frequency, or phone call duration) showed any effect on caries progression between baseline and the 1- or 2-y follow-ups (Table 3).

**Table 3.** Univariable Logistic Regressions of the increase in Total Observed Caries Experience (TOCE) and in New Caries at the Surface Level at the 1- and 2-y Follow-ups Compared to Baseline, on Toothbrushing Habits (at 1-y Follow-up Only), Parental Background, Intervention Language, Phone Call Frequency, or Phone Call Duration, in the Intervention Group.

	1-y Follow-up versus Baseline			2-y Follow-up versus Baseline		
Variables	OR	95% CI	*P* Value	OR	95% CI	*P* Value
Increased TOCE						
Toothbrushing 1× daily	1.78	0.48–6.55	0.387	—		
Toothbrushing 2× daily	1.58	0.47–5.23	0.458	—		
Parental background^a^						
Europe (outside Sweden)	0.60	0.09–4.17	0.605	0.38	0.03 – 4.55	0.446
Outside of Europe	1.13	0.19–6.70	0.897	1.93	0.15 – 24.46	0.612
Intervention language^b^						
Swedish native (Swedish)	1.24	0.20–7.67	0.821	0.39	0.02 – 7.11	0.524
Not Swedish native (native language)	0.91	0.25–3.25	0.882	0.24	0.02 – 2.33	0.217
Phone call frequency	1.00	0.87–1.15	0.997	1.00	0.82 – 1.22	0.990
Phone call duration	1.00	0.99–1.01	0.568	1.01	0.99 – 1.02	0.511
New caries at surface level						
Toothbrushing 1× daily	2.36	0 62–8.99	0.208	—		
Toothbrushing 2× daily	1.11	0.32–3.86	0.865	—		
Parental background^a^						
Born in Europe (outside Sweden)	1.22	0.16–9.47	0.848	0.25	0.02–2.84	0.263
Born outside of Europe	1.13	0.19–6.70	0.897	0.32	0.03–2.98	0.315
Intervention language^b^						
Swedish native (Swedish)	0.56	0.10–3.05	0.499	1.75	0.28–11.15	0.554
Not Swedish native (native language)	1.11	0.28–4.48	0.882	1.87	0.47–7.35	0.372
Phone call frequency	1.06	0.92–1.23	0.409	0.97	0.84–1.12	0.627
Phone call duration	1.00	0.99–1.01	0.938	1.00	0.99–1.01	0.611

CI, confidence interval; OR, odds ratio; TOCE: Total Observed Caries Experience.

a Based on reported native language, reference: Sweden.

b Reference: not Swedish native, intervention in Swedish.

### Caregiver Self-Reported Importance and Confidence

Caregivers in the intervention group reported high but decreasing levels of perceived importance over time with a change in mean score from 9.1 to 8.7. Mean score for confidence improved from 7.8 to 8.6, pre- to postintervention (Appendix Figure 1).

## Discussion

Despite implementing intensive preventive support measures in the year following surgery, we observed high rates of caries relapse, higher than reported in other studies (Amin et al. 2010; Jiang et al. 2019; Zhao et al. 2022). At the 2-y follow-up, however, recurrence was in line with previous reports ranging from 53% to 79% (Almeida et al. 2000; Foster et al. 2006; El Batawi 2014). Our intervention group began with a higher total caries burden at baseline and, despite improved dietary habits, continued to develop significantly more caries over time. A previous prevention study also targeting children with a severe caries burden reported similar findings with a caries prevalence >90% after 3 y and a higher increment in the intervention group (Braun et al. 2016), highlighting baseline caries experience as an important predictor of future caries development (Mejare et al. 2014).

One explanation for the lack of intervention effects may be the implementation. We designed our intervention to occur biweekly over the course of a year, yet only 6 families engaged in at least 20 sessions. Participants contributed to determining and agreeing on the call schedule, reflecting a collaborative approach (AAPD 2013) that respected their autonomy, ensured informed consent, and maintained feasibility. Imposing a nonnegotiable biweekly call schedule might have enhanced scientific validity. Balancing scientific rigor with respect for participants’ rights and well-being is essential. The smaller sample size, compared with the calculated requirement, may also have resulted in insufficient statistical power to detect an effect. Previously low parental adherence to oral health programs has been reported for high-risk caries groups (Braun et al. 2016). This may reflect parental perception of the severity of the caries disease, low intervention value, or the competing demands of everyday life in a family (Albino et al. 2018; Batliner et al. 2018).

The OHCs received training and supervision to ensure adherence to MI principles and the intervention protocol. Still, lack of proficiency in MI among the OHCs may partially explain our result. Few studies on MI fidelity in caries prevention have been published (Jamieson et al. 2016; Weinstein et al. 2014; Wilson et al. 2018), and conflicting associations have been reported for caries-related outcomes (Wilson et al. 2018). In this study, we used MITI 4.1 for self-reflection, but we did not collect the results for study purposes. This is a limitation because it restricts our ability to assess whether MI was implemented as intended. Data on MI fidelity would have strengthened our evaluation and provided deeper insights into its impact on the study outcomes. However, there are currently no validated tools for assessing threshold proficiency in dental settings (Leske et al. 2021). Reported challenges in health talks with patients include cultural barriers, environmental distractions, and counselors resorting to generic advice (Leske et al. 2021). While repeat follow-up phone calls has previously been proven useful (Jiang et al. 2020) and cost-efficient (Pukallus et al. 2013) in caries prevention, our reliance on this contact path alone may have increased the challenges compared with face-to-face interactions. A strength of our study was the multilingual competence; however, 38% of the intervention group did not receive support in their native language, which may have posed a barrier.

The lack of effect may also be due to the intervention being ineffective. Behavior-change approaches focusing on ECC, including the MI approach, have mixed results (Albino and Tiwari 2016). The conflicting results can be partly explained by methodological differences, where reports on the necessary number, duration, and time span of the support required to prevent caries vary. The present study found no relationship between intervention intensity and caries outcomes. Our results are in line with other recent studies reporting that when targeting high-risk populations, oral health promotion (Braun et al. 2016) as well as MI (Batliner et al. 2018; Henshaw et al. 2018) are not effectively preventing future decay. Several studies reporting positive results with MI have targeted caregivers with oral health promotion during pregnancy (Jamieson et al. 2016) or infancy (Weinstein et al. 2006; Harrison et al. 2007; Colvara et al. 2018), rather than supporting parents of children who have already established severe caries disease, as in this study. To effectively reduce ECC, it may be important to distinguish between the various stages of prevention. Once established, the disease trajectory of caries underscores the importance of early intervention rather than trying to manage downstream factors.

Disadvantaged socioeconomic circumstances, including parental income, education, and cultural background, increase the susceptibility of children to dental caries (Fisher-Owens et al. 2007; Schwendicke et al. 2015). Neither residential area nor parental background emerged as significant caries predictors in our material. However, parental background was based on reported native language for counseling, and residential area was considered at the group level. Not collecting more granular socioeconomic data limited our ability to analyze the effect of such influence. Given that more than half of the primary teeth, on average, were affected by caries at baseline in this study, the high disease burden may also reflect a family context with cumulative psychosocial stressors and limited resources for prioritizing oral health (Albino and Tiwari 2020; Braun et al. 2016). Previous studies suggest that even when parents acquire more knowledge, behaviors and caries outcomes are not always changed (Braun et al. 2016; Henshaw et al. 2018). In our study, the overall high scores for motivation to change, together with the distressing caries rates, might suggest an “intention-behavior gap,” that is, parental intentions to support the oral health of their child do not always translate into required behaviors for halting caries progression (Sheeran et al. 2016). Variables that initiate and sustain behavior change, as well as upstream factors’ effect on current behaviors, need further attention (Albino and Tiwari 2020).

## Conclusion

Within the limitations of this study, while the parental support program offering oral health coaching via telephone influenced dietary habits, no significant effect on caries progression was observed in preschool children with severe ECC. These findings offer valuable insights into intervention strategies for high-risk children. Effective ECC prevention will likely need interventions targeting upstream factors alongside individual approaches, demanding innovative interdisciplinary methods.

## Author Contributions

I. Brännemo, contributed to conception, design, data acquisition, analysis, and interpretation, drafted and critically revised the manuscript; T. Hasselblad, contributed to conception, design, critically revised the manuscript; A. Levinsson, contributed to data analysis and interpretation, drafted and critically revised the manuscript; G. Dahllöf, contributed to conception, design, data interpretation, critically revised the manuscript; G. Tsilingaridis, contributed to conception, design, data acquisition and interpretation, critically revised the manuscript. All authors gave their final approval and agreed to be accountable for all aspects of the work.

## Supplemental Material

sj-pdf-1-jct-10.1177_23800844241296054 – Supplemental material for Phone-Based Parental Support Program for Caries Prevention in Children: A Randomized Controlled Trial Supplemental material, sj-pdf-1-jct-10.1177_23800844241296054 for Phone-Based Parental Support Program for Caries Prevention in Children: A Randomized Controlled Trial by I. Brännemo, T. Hasselblad, A. Levinsson, G. Dahllöf and G. Tsilingaridis in JDR Clinical & Translational Research
