# Supplementary material for: Phone-Based Parental Support Program for Caries Prevention in Children: A Randomized Controlled Trial
Source: JDR Clin Trans Res. 2024 Dec 4;10(3):304–14. doi: 10.1177/23800844241296054 (PMC12166145; doi:10.1177/23800844241296054)

Appendix

JDR Clinical & Translational Research

Phone-Based Parental Support Program for Caries Prevention in Children: A Randomized Controlled Trial

Ida Brännemo[1,2], Tove Hasselblad[1], Anna Levinsson [3,4,] Göran Dahllöf[1,2,5], Georgios Tsilingaridis[1,2]

[1]Division of Pediatric Dentistry, Department of Dental Medicine, Karolinska Institutet, Stockholm, Sweden

[2]Center for Pediatric Oral Health Research, Stockholm, Sweden

[3]Department of Epidemiology, Biostatistics, and Occupational Health, McGill University, Montreal, QC, Canada

[4]Department of Social Medicine and Public Health, Sahlgrenska Academy, Gothenburg University, Gothenburg, Sweden

[5]Center for Oral Health Services and Research Mid-Norway, TkMidt, Trondheim, Norway

Corresponding author:
Ida Brännemo
Division of Pediatric Dentistry
Department of Dental Medicine
Karolinska Institutet
SE-141 86 Huddinge, Sweden

Phone: +46-8-524 880 39
E-mail: ida.brannemo@ki.se

Appendix

Appendix Table 1a. Negative Binomial Generalized Estimating Equation Models for the Association Between Total Observed Caries Experience (TOCE) and Group Over Time

TOCE	Main effects			Main effects and interaction term		
	IRR	95% CI	*p*-value	IRR	95% CI	*p*-value
Group (Intervention vs control)	1.06	0.98 – 1.16	0.169	1.03	0.92 – 1.15	0.652
Time (1-year follow-up vs baseline)	1.09	1.08 – 1.11	< 0.001	1.08	1.06 – 1.11	< 0.001
Interaction group*time				1.02	0.99 – 1.05	0.231

Note. CI, confidence interval; IRR, incidence rate ratio

Appendix Table 1b. Logistic-Generalized Estimating Equation Models for the Association Between Tooth brushing Habits and Group Over Time

	Main effects			Main effects and interaction term		
	OR	95% CI	*p*-value	OR	95% CI	*p*-value
Tooth brushing 1x daily						
Group (Intervention vs control)	0.98	0.55 – 1.73	0.939	1.09	0.64 – 1.88	0.751
Time	2.53	1.58 – 4.07	< 0.001	1.39	1.13 – 1.72	0.002
Interaction group*time				0.94	0.70 – 1.26	0.672
Tooth brushing 2x daily						
Group (Intervention vs control)	1.09	0.63 – 1.88	0.770	1.47	0.38 – 5.67	0.580
Time	1.70	1.11 – 2.60	0.014	1.88	1.02 – 3.46	0.042
Interaction group*time				0.81	0.35 – 1.90	0.634

Note. CI: confidence interval; OR: odds ratio

Appendix

Appendix Table 2. Univariable Logistic Regressions of Increase in Total Observed Caries Experience (TOCE) and New Caries at the Surface Level (at the 1- and 2-Year Follow-Ups) on Group, Tooth brushing Habits (at the 1-Year Follow-Up) and Parental Background.

	1-year follow-up vs baseline			2-year follow-up vs baseline		
	OR	95% CI	*p*-value	OR	95% CI	*p*-value
Increase in TOCE						
Group (Intervention vs control)	1.97	0.91 – 4.27	0.087	3.60	1.29 – 10.05	0.015
Tooth brushing 1x daily	1.16	0.47 – 2.84	0.750	-		
Tooth brushing 2x daily	1.29	0.60 – 2.78	0.518	-		
Parental background*						
Europe (outside Sweden)	0.46	0.13 – 1.61	0.221	0.67	0.18 – 2.54	0.556
Outside of Europe	0.59	0.19 – 1.79	0.350	1.56	0.46 – 5.24	0.474
New caries at surface level						
Group (Intervention vs control)	1.82	0.81 – 4.07	0.147	2.17	0.95 – 4.94	0.065
Tooth brushing 1x daily	2.09	0.85 – 5.18	0.110	-		
Tooth brushing 2x daily	1.46	0.65 – 3.29	0.363	-		
Parental background*						
Europe (outside Sweden)	0.80	0.23 – 2.76	0.724	0.79	0.23 – 2.70	0.711
Outside of Europe	1.04	0.35 – 3.06	0.943	1.42	0.49 – 4.09	0.518

Note. *: based on reported native language, reference: Sweden
CI: confidence interval; OR: odds ratio; TOCE: total observed caries experience.

Appendix

Appendix Figure 1. Importance and confidence scales over the first 12 months posttreatment in the intervention group. Importance items assess participant perceptions of "how important" it is that they engage in recommended parental oral health behaviors while confidence items assess "how sure" they feel about engaging in such behaviors. For both, responses were measured on a 10-point scale: 1 = not at all, 10 = very much.

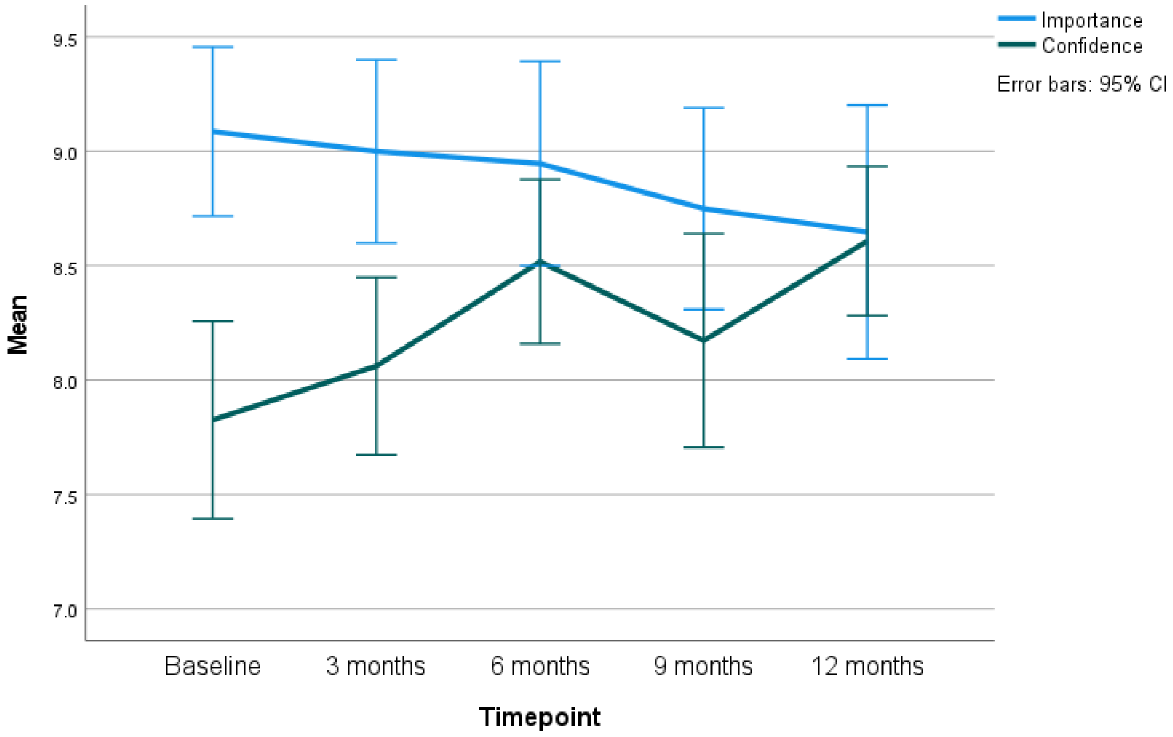

Supplement: sj-pdf-1-jct-10.1177_23800844241296054 – Supplemental material for Phone-Based Parental Support Program for Caries Prevention in Children: A Randomized Controlled Trial [file sj-pdf-1-jct-10.1177_23800844241296054.pdf]
